# A national survey on COVID-19 infection in Italian retirement homes for older adults and persons with disabilities

**DOI:** 10.3389/fpubh.2026.1872101

**Published:** 2026-06-30

**Authors:** Alice Paggetti, Sara Maria Pani, Patrizia Lorenzini, Emanuela Salvi, Enrica Vignaroli, Virginia De Silva, Gilda Losito, Ciro Tarantino, Nicola Vanacore, Domitilla Marconi, the Italian RHOAD Study Group, Abrate Mauro, Agati Salvatore, Aiello Alessandro, Albanese Maria, Aldeghi Luca, Alessio Laura, Alvisi Giuliano, Ambrosio Ilaria, Angelino Remo, Arcangeli Lino, Ardu Alessandra, Arrobbio Cristiana, Arru Sara, Arturi Serena, Ascheri Lorenzo, Astegiano Roberto, Avantaggiato Clara, Baesso Antonio, Balbo Bruno, Balbo Viola, Banfi Mirco, Barba Calogero, Barbara Bergo, Barbero Valeria, Barbieri Virna, Barone Gianmarco, Barzaghi Alberto, Basile Roberta, Beccaria Antonella, Bellini Maria Saveria, Benigni Luca, Benini Davide, Benzi Maria Rita, Bernardello Davide, Bernardi Katia, Berta Barbara, Berton Mirella, Bertossi Cristina, Bertuletti Giuseppe, Besutti Raffaele, Bianco Roberta, Binni Giacomo, Biografi Arianna, Bisconti Giovanni, Blanch Lorena, Bocca Daniela, Bocchini Cristina, Bogani Valentina, Boggio Barbara, Boggio Carla, Boldori Gianpaolo, Bolondi Fabrizio, Bolzoni Laura, Bonato Simona, Bonetti Maria Erika, Boni Ilaria, Bonvicini Flaviano, Boreggio Mario, Borgna Franco, Borraccia Valentina, Borroni Silvia, Borsati Angelina, Bortolin Carmela, Boscaro Federica, Boschetti Vittorio, Bosco Luca, Boscolo Fiore Elena, Bosi Andrea, Bosio Stefano, Bottazzoli Federico, Botter Cinzia, Botticella Maria Antonia, Bottone Daniela, Bozzato Elisabetta, Brach Loieta Laura, Braghin Luisa, Bragion Mariarosa, Braida Rossella, Brancato Giuseppe, Brasile Raffaele, Brianti Giancarlo, Briccolani Debora, Brugnoli Giulia, Bruno Bianca, Bruno Fulvio, Bruno Lorenza, Bruno Luciano, Bruno Sandro, Buccarella Valentina, Bulf Bruno, Bulgari Francesca, Bulleri Mariella, Buongiorno Adriana, Buoso Luca, Cacciatori Stefano, Cafaro Ludovico, Caimmi Michele Joris, Caldera Simona, Calicchio Giuseppe Nicola, Camilli Meletani Serena, Campanale Vito, Candela Giuseppe, Cannavacciuolo Andrea, Cannito Francesco, Capo Giuseppe, Cappannini Vincenzo, Cappelli Caterina, Capriotti Ines, Caramanico Luisa, Carlino Matteo, Carnuccio Anna Maria, Caruso Mariacarmela, Casalone Rinaldi Marta, Castagna Ilaria, Castoldi Davide, Catalano Ravaglioli Marta, Cattanei Mauretta, Cavion Barbara, Celentano Chiara, Cencetti Alba, Ceresa Paola Giuseppina, Chianese Valentina, Chiaradia Giuseppe, Chiolo Luigi, Chittaro Loris, Cinelli Cinzia, Ciocchini Valentina, Cioni Sara, Cocco Chiara, Colamaio Giuseppe, Colombino Roberta, Colombo Lisa, Colombo Sonia, Colturi Gennj, Coppola Cristina, Cornaglia Paola, Corsano Valentina, Corsaro Maria, Covatta Maria Vittoria, Craviolo Chiara, Crispino Giorgio, Cucchio Simona, Curti Luisa, Dagani Erika, Dal Mas Stefano, Danzi Donatella, De Caris Rita, De Dea Valeria, De Faveri Piero, De Leo Ilaria, De Maria Mira M.T., De Nale Paola, De Pieri Veronica, De Rosa Massimo, Debenedetti Federica, Degiovanni Silvano, Degl'Innocenti Marcello, D'Elia Carlo, Desogus Cesare, Di Cristofaro Carmelina, Di Dolco Francesco, Di Fazio Vincenzo, Di Pietro Luisa, Di Santo Daniela, Di Stefano Chiavaro Vita, Dogliani Maura, Donati Silvia, Dotta Federica, Dotti Manuela, Dubinin Pietro Antonio, Duretto Cristina, Egidio Rodolfo, Ena Andrea, Enrico Gianluca, Fabbrica Debora, Facci Francesco, Fariello Francesco, Fenile Massimo, Fera Achille, Feroldi Zaira, Ferracin Roberta, Ferrante Alfredo, Ferrara Angelina, Ferrara Vito, Ferrari Massimo, Ferrari Michele, Ferrario Fabio, Fino Stefania, Fiorentini Samantha, Flaccadori Emanuele, Fontana Solange Dominique, Formentin Roberta, Fornaro Franca, Franchi Patrizia, Frigo Beatrice, Gabaldo Fabrizio, Gaiardoni Carlo, Galaschi Donatella, Galletta Giulia, Gallon Dario, Galluzzo Vincenza Cinzia, Gamberini Maria Giacinta, Garzola Elena, Genetti Cristian, Gentile Simona Carmen Alessandra, Ghetti Greta, Ghio Daniela, Ghio Giacomo, Ghisellini Simona, Giachino Daniela, Giacon Marco, Giannoni Federica, Giaretta Anna, Giletta Giuseppe, Gillardo Mirko, Ginocchi M.Luisa, Giurca Gabriela Cristina, Gorga Caterina, Gori Maria Cristina, Gottardello Piera Antonia, Grandini Alessandro, Grasso Elisabetta, Gravante Rosanna, Graziani Manuela, Grazzini Cristina, Greco Giovanni, Greco Maria, Greco Rito, Gregnanin Vanja, Grigolon Lorenzo, Groppelli Marta, Guidotti Lucia, Ianniello Giovanna, Iannolo Sara, Iannone Rosa, Iemmi Stefania, Kutin Anna, Laganà Armenia, Lalli Vincenzo, Lampreu Agnese, Lanciotti Fabrizio, Landi Lara, Lavatori Paolo, Leone Nicoletta, Leoni Oriano, Leta Serena, Liberati Mario, Linguanti Sonia, Lombardi Paola, Lombardi Vincenzo, Loni Veronica, Lorello Anna Elvira, Lucarini Patrizia, Luciano Paola, Lulleri Sabrina, Lupetti Ivana, Madonia Carmelo, Madrigali Marta, Magani Fabrizio, Magni Sara, Maiello Diletta, Malagò Pier Maria, Malinverni Pierpaolo, Mammola Aldo, Mancinelli Elia, Mancini Maria Luisa, Manfredi Francesco, Manoni Fiorella, Marastoni Matteo, Marceca Ivana, Marchetto Roberta, Maresca Antonino, Maria Ferrari, Mariani Gaetana, Marini Cecilia, Martineli Giulia, Martini Maria, Masala Daniela, Masini Arturo, Mason Silvana, Massaro Clara, Mastrodomenico Stefano, Matina Antonio, Matta Cecilia, Maurandi Giancarlo, Mauro Orietta, Mazzanti Monia, Mazzuca Mariaclaudia, Mazzucchelli Sara, Meggiolaro Gianmario, Melenzio Nella, Memoli Loredana, Menini Fabio, Mercantile Maria Luisa, Merella Waltere, Merletti Sergio, Merra Salvatore, Mesiti Rosa, Messori Elena, Mezzadra Davide, Mezzadra Valeria, Miccichè Giuseppe, Mihalache Ana Maria, Milano Brunello, Millo Eva, Mina Katia, Mingrone Rosa, Mondino Marco, Mora Romina, Morcelli Valentino, Moretti Lucia, Morici Moreno, Morrone Marco, Moscato Brunetta, Motta Santo, Munari Diego, Muraca Luciano, Mussner Erna, Nappi Carmine, Nasuti Emilio, Nicolò Costantino, Nicosia Maria, Niedermayr Kurt, Nisticò Maria, Nizzoli Silvia, Notari Cleide, Novelli Andrea, Nuti Federica, Oliverio Martina, Orlandi Antonella, Ortolan Dania, Pagliai Costanza, Paialunga Ivana, Palermo Mario, Palmerio Vincenzo, Palmieri Carla, Panu Ester Vittoria, Panzacchi Giacomo, Paolo Battocchio, Pappacena Francesco, Patella Luigi, Paternò Lidia, Pecoraro Roberto, Perina Valter, Perna Alfonso, Peruzzo Simona, Piazza Pierluigi, Piccinni Liliana, Pina Francesca, Pinotti Marica, Piscopo Anna, Pistillo Mirella, Pituello Matteo, Pizzo Elisabetta, Poggio Cinzia, Poloni Daniela, Pompili Giuseppina, Poppi Maura, Pozzobon Elisa, Prati Alfonso, Preiata Samuele, Prezioso Alessandro, Puddu Laura, Punnakkezhath Victoria, Quadretti Fabio Romano, Quondam Girolami Donatella, Racalbuto Giovanni, Ragaglia Giampaolo, Ramacciotti Daniele, Ramello Cinzia, Rao Donatella, Rendina Enrico, Riggi Dalila, Rizzo Tiziano, Roasio Silvano, Rocco Lorella, Rognoni Suor Natalina, Romanò Stefano, Romoli Vanessa, Rossetti Maria Pia, Rossi Giorgio, Rubin Lucia, Rudella Lorenzo, Ruffo Valentina, Rusu Meluta Ecaterina, Saba Giorgia Valeria, Sacchi Erica, Said Siwar, Salvicchi Rodolfo, Sandri Moira, Santesso Paolo, Santoianni Alessandro, Saraino Claudio, Sartor Rosetta, Sassano Francesco, Sasso Flora, Scavone Vincenzo, Schiavon Davide, Sciacione Silvia, Scirocco Giovanna, Sciumbata Valentina, Scomparin Fabrizio, Sconosciuto Antonio, Scotti Giovanni, Scranni Simone, Sedda Sara, Segato Mauro Antonio, Senatori Silvia, Servodio Valentina, Settembrini Vincenzo, Setti Alessio, Sibilla Salvatore, Sicilia Antonio, Signorile Massimiliano, Simon Simon Jesy, Sironi Stefano, Soave Daniela, Sora Elena, Spadone Raffaella, Stanga Valentina, Stecco Francesca, Stefanin Cristina, Stortolani Stefano, Succu Mariaflora, Taccardi Riccardo, Tacchino Di Girolamo Emanuela, Tardino Rosa Maria, Tardivo Marta, Thomas Anneyamma, Titon Fabrizia, Tiziani Tiziana, Todaro Biagio, Tola Rita Erina, Toniolo Alberto, Tonon Monica, Toppano Paola, Torti Claudia, Tosi Silvia Tosi Silvia, Trattenero Chiara, Trezzolani Alessandra, Tronchin Marco, Trucchi Giuseppe, Tuninetto Claudio, Uliana Maria Aurora, Vadori Marcello, Valdambrini Elisa, Valentini Arnaldo Rodolfo, Valori Virna, Vandini Desolina, Vanzetti Adele, Vazzana Angelo, Veggetti Marina, Venturini Nicole, Verdolin Dino, Vigato Federico, Vignoli Massimiliano, Vignoli Valeria, Visigalli Andrea, Vitali Nicoletta, Vito Giuseppe, Vottero Sergio, Zago Paola, Zambelli Angela, Zampicinini Franco, Zanca Pamela e Merli Federica, Zanini Annalisa, Zannoni Daniela, Zanotti Luigi, Zanzanaini Paola, Ziella Jonathan, Zigliani Roberta, Zingale Valeria, Zoccarato Cinzia

**Affiliations:** 1 National Center for Disease Prevention and Health Promotion, Italian National Institute of Health, Rome, Italy; 2 Department of Medical Sciences and Public Health, University of Cagliari, Cagliari, Italy; 3 National Center for Drug Research and Evaluation, Italian National Institute of Health, Rome, Italy; 4 Italian National Guarantor for the Rights of Persons Deprived of Liberty, Rome, Italy; 5 CRS– Service for Skills Development and Social Responsibility, Central Directorate for Human Resources, Italian National Institute of Statistics (ISTAT), Rome, Italy; 6 University of Perugia, Perugia, Italy; 7 Independent Researcher, Rome, Italy; 8 Public Prosecutor's Office of Brescia – Department of Judicial Organization, Personnel, and Services, Ministry of Justice, Brescia, Italy; 9 Department of Humanities, University of Calabria, Cosenza, Italy; 10 Department of Educational Sciences, Psychology, and Communication, Suor Orsola Benincasa University of Naples, Naples, Italy; 11 Post Graduate School of Public Health, University of Siena, Siena, Italy

**Keywords:** care homes, COVID-19, disability, long-term care, older people, preparedness

## Abstract

**Introduction:**

Older people and individuals with chronic illnesses living in long-term care facilities are at increased risk of infections due to the care setting, resulting in an increased probability of contracting SARS-CoV-2 and experiencing severe sequelae during the COVID-19 pandemic. To limit viral transmission, facilities adopted different policies according to national and regional prevention strategies, often reducing social contacts and promoting public health measures. We therefore conducted a national survey to investigate the impact of COVID-19 infection in Italian retirement homes for older adults and persons with disabilities (RHOAD).

**Methods:**

An online questionnaire investigating facility characteristics and COVID-19 impact between March 2023 and January 2024 was sent via email to Italian RHOAD.

**Results:**

Response from 510 RHOAD showed a trend in the percentage of COVID-19-positive individuals similar to that observed in Italy. An increase in cases was recorded in 2020, followed by a gradual decrease in 2021. Specific facility characteristics, such as a larger number of beds (80 or more vs. 0–20, OR 2.97, 95%CI 1.10–8.03), or challenges in transferring (OR 11.79, 95%CI 3.32–41.91) or isolating residents with COVID-19 (OR 4.43, 95%CI 2.13–9.22) or geographical location in the South (OR 0.17 95%CI 0.07–0.39) or Center (OR 0.39 95%CI 0.16–0.93) vs. North, were associated with an increased risk of having at least one COVID-19 case among residents in the multivariable logistic regression analysis. Similarly, facilities reporting at least one COVID-19 case applied national prevention measures related to visitor access less strictly (OR 2.16 95%CI 1.06–4.38) and registered an increase of adverse events (OR 2.49 95%CI 1.17–5.27).

**Discussion:**

Our findings highlighted the challenges faced by Italian RHOAD during the pandemic and support the optimization of the RHOAD organization to prevent disease transmission.

## Highlights

- Italian long-term care facilities mostly host non-self-sufficient individuals.
- COVID-19 in long-term care facilities mirrored the Italian geographical spread.
- Long-term care facility features are linked to COVID-19 spread risk among residents.
- Adverse events, restraints, and psychotropics in facilities rose during the pandemic.

## 1 Introduction

COVID-19, caused by the SARS-CoV-2 virus, is an acute respiratory infection primarily affecting the lower respiratory system. Although immunocompetent individuals typically experience mild symptoms or remain completely asymptomatic, some subjects may develop severe disease, particularly vulnerable populations such as older adults and people with chronic diseases. The adverse progression of severe symptoms may lead to life-threatening complications and death (1, 2). Furthermore, older adults and individuals with chronic illnesses living in long-term care (LTC) facilities are at increased risk of infections due to the characteristics of these settings. Indeed, sharing of living spaces with other residents, the presence of staff members who often work across multiple facilities (hospitals, clinics, other LTC facilities), and visits from relatives and friends significantly heighten the likelihood of outbreaks of infectious diseases such as COVID-19 (3, 4). There are various types of LTC facilities, which differ according to the needs they are designed to meet and the social and healthcare organization of the country in which they are located. The National Institute on Aging (5) offers a guide to the main categories of LTC facilities (see Table 1). In the present work, we focused on a specific type of LTC facility in Italy, which combines characteristics of residential care homes and assisted living facilities, namely retirement homes for older adults and persons with disabilities (RHOAD). These facilities primarily provide social and rehabilitative support to adults with varying levels of disability and do not offer the same degree of continuous medical and nursing care as nursing homes.

**Table 1:** Main categories of Long-Term Care (LCT) facilities according to the National Institute of Aging.

Main categories of LCT facilities	
Category	Definition
Residential care home (Board and care homes) or Group homes	Small private care facilities that do not provide health care services.
Nursing homes (skilled nursing facilities)	Offer a wide range of health and personal care services focused primarily on medical care and supervision throughout the day.
Assisted living facilities	Dedicated to those who need help with daily care but not as much as nursing home residents.
Continuing care retirement communities (life care communities)	Offer various levels of services among those mentioned above in a single location.

In these facilities, which may vary in size and number of residents, older adults, and people with disabilities can live while receiving support with activities of daily living. In Italy and, more broadly, across Europe, people living in LTC facilities are on average over 80 years old (6), and often present conditions that make them particularly vulnerable from both mental and physical health perspectives (7–9). Moreover, it has been documented that about 60% of them live with dementia (6, 10–13). These aspects, which are often insufficiently emphasized, are extremely relevant because dementia is frequently associated with a greater psychophysical vulnerability and an increased need for care provided by trained professionals (14–17). These needs often cannot be fully met by LTC facilities, which, by their nature, are not always healthcare settings and may therefore lack the staffing levels and resources required to care adequately for severely ill patients or those isolated due to COVID-19 infection (18, 19).

The Italian National Recovery and Resilience Plan (NRRP) devotes great attention to improving the living conditions and social inclusion of older adults and non-self-sufficient individuals. Several actions aim to promote more home-like and less institutionalized environments, enhancing home-care services (Mission 5) (20), integrating hospital and community services, and encouraging multi-professional and interdisciplinary approaches to foster person-centered care and continuity of care (Mission 6) (20). The difficulty experienced during the pandemic in accessing personal protective equipment (PPE) and other measures needed to contain the spread of SARS-CoV-2 (7, 21) further increased health risks for both residents and staff in LTC facilities. These aspects clearly highlight the critical situation faced by these settings during the pandemic. Indeed, Europe and the United States reported very high numbers of deaths in LTC facilities. In particular, in Europe, deaths among LTC residents accounted for 37%−66% of all COVID-19-related deaths during the first wave of the pandemic (March–May 2020). Italy was the first Western country to be severely affected by the COVID-19 outbreak, with the Lombardy region (Northern Italy) reporting the highest mortality rate in LTC facilities in Europe during the first wave (22–24).

Therefore, to limit the spread of SARS-CoV-2, many LTC facilities adopted a range of measures, including very strict restrictions on visitor access, social distancing policies, education on PPE use, and staff training in infection control (8). Moreover, each country adopted its own strategy to contain the pandemic, often relying on isolation and reduction of social contacts, as well as the promotion of public health measures (25). In Italy, the first response to COVID-19 consisted of a national lockdown from March to May 2020, as established by the Decree of the President of the Council of Ministers (DPCM) of 9 March 2020. Thereafter, restraint measures were loosened or tightened based on regional and local epidemiological trends. On the 8th of May 2021, the DPCM regulated access to and departure from LTC facilities for residents and visitors, limiting opportunities for virus transmission. These regulations could also be applied more restrictively at the discretion of the competent health authority based on the local epidemiological context.

Although these measures aimed to limit the spread of infection and protect vulnerable populations, social isolation may have contributed to reduced mental wellbeing (26) and increased negative emotional and behavioral events (27). In this regard, following visits to two Italian nursing homes in 2022, the European Committee for the Prevention of Torture and Inhuman or Degrading Treatment or Punishment expressed concern about the ongoing restrictions in place since February 2020. The committee highlighted that limited access to outdoor areas, reduction of rehabilitation and recreational activities, and restriction on family visits had progressively deleterious effects on both the mental and physical health of residents, considering this situation a form of deprivation of liberty (28).

Previous works have investigated the impact and management of the COVID-19 pandemic in Italian LTC facilities, reporting high mortality rates among residents infected with SARS-CoV-2 and identifying organizational characteristics associated with increased risk of COVID-19 outbreaks, such as staff shortages, difficulties in isolating residents or transferring them to other facilities, as well as larger facility size and higher number of beds (29, 30). Previous studies, investigating mortality, outbreak occurrence, and structural risk factors (31, 32), mainly focused on nursing homes and LTC settings that provide higher levels of medical and nursing care than RHOAD. As a result, limited evidence is available on less medicalized residential settings such as Italian RHOAD, which differ substantially in their organizational model, staffing patterns, level of healthcare provision, and resident case-mix. Moreover, previous research has not examined how these facilities managed infection-control measures, ensured continuity of daily care, and addressed operational challenges during the different phases of the pandemic. Nor has it explored setting-specific vulnerabilities and protective factors that may have influenced pandemic outcomes among residents with complex cognitive and functional impairment. This represents an important gap in knowledge, as findings from nursing homes and more healthcare-oriented LTC settings may not be directly transferable to RHOAD. To our knowledge, the present study, conducted by the Dementia Observatory of the Italian National Institute of Health (33), in collaboration with the Italian National Guarantor for the Rights of Persons Deprived of Liberty, is the first to investigate the impact and management of the COVID-19 pandemic in the Italian RHOAD. By focusing on a largely neglected residential setting that hosts vulnerable populations, this study aims to address a significant gap in the literature.

Specifically, the study aims to: (i) assess the spread of SARS-CoV-2 infection among residents and staff in RHOAD during the different phases of the pandemic, (ii) evaluate the organizational responses adopted by facilities, including infection-control practices, management of suspected and confirmed cases, and changes in routine care, (iii) examine the operational challenges encountered during the emergency, such as insufficient or unclear information regarding infection-control procedures and barriers to effectively isolating suspected or confirmed COVID-19 cases, and (iv) identify facility-level vulnerabilities and protective factors that influenced pandemic outcomes.

## 2 Methods

### 2.1 Questionnaire

We conducted a national survey of the Italian RHOAD through an online questionnaire (see Additional File 1). The questionnaire was developed by a multidisciplinary group based on previous research on nursing homes during the COVID-19 pandemic carried out by the OssDem-ISS in 2020 (29, 30). Each facility received its own credentials to access the questionnaire, and the director of each facility was responsible for filling it out. Data were collected through a web-based platform and saved in a database.

The questionnaire consisted of two main sections. Section 1 collected information on the characteristics of the facility at the time of completion, including the type of structure (public or providing services both privately and within the public social system) and resident characteristics. Section 2 aimed to collect aggregated data on the COVID-19 pandemic, including issues and challenges faced by the facilities. This section explored the spread of the infection and its outcomes (number of hospitalizations and deaths), the infection prevention and control program and practices adopted, and the flu and COVID-19 vaccination status of residents. All questions related to the COVID-19 pandemic referred to the period from 1 February 2020 to 31 December 2021.

### 2.2 Data source

The Italian National Guarantor for the Rights of Persons Deprived of Liberty (https://www.garantenazionaleprivatiliberta.it/gnpl/) provided the complete list of 5,877 long-term care facilities available and accredited in Italy. We excluded 2,006 facilities because they were nursing homes, facilities for minors, duplicates, or facilities no longer active in 2023. The distinction was made based on the official regional accreditation categories reported in the Guarantor's registry, which allowed us to separate RHOAD from other facilities. This distinction is relevant because both the baseline health status of residents and the organizational characteristics (e.g., presence of medical personnel, capacity for isolation, infection-control protocols) differ between the facility types, potentially influencing COVID-19 risks and management strategies.

Between March 2023 and January 2024, we sent the questionnaire by email, accompanied by a cover letter, to all 3,871 Italian RHOADs, corresponding to 120,189 beds. We also contacted by phone (five operators made approximately 800 calls) most of the facilities previously reached via email (an initial invitation, followed by two follow-ups in the following months) to renew the invitation to participate and offer assistance when needed.

### 2.3 Statistical analysis

For statistical analysis, data were extracted from the database and underwent quality and consistency checks. A descriptive analysis was performed using frequencies and percentages for categorical variables and means with ranges or medians with interquartile ranges for continuous variables.

The characteristics of facilities that completed the second section of the questionnaire were compared with those that did not, in order to assess potential imbalances between the two groups.

The statistical analysis focused on the characteristics of the facilities according to whether they reported at least one confirmed case of COVID-19 infection during the study period. The outcome of the analyses was defined as the occurrence of at least one COVID-19 case, since even a single case may be considered an outbreak because of the substantial public health consequences associated with this infection, in accordance with the scientific literature (32, 34).

The definition of a confirmed COVID-19 case follows the definition provided by the Italian Ministry of Health, in accordance with WHO and ECDC guidance, as an individual meeting laboratory diagnostic criteria for SARS-CoV-2 infection (35–37). Although these criteria evolved over time in response to the changing epidemiological scenario, they were applied uniformly at the national level, ensuring comparability across facilities at each time point.

Comparisons between facilities that reported at least one case and those reporting no cases were performed using the chi-square test for categorical variables and the Mann-Whitney test for continuous variables. Univariable and multivariable logistic regression analyses were conducted to assess the characteristics of the RHOAD associated with COVID-19 outbreaks.

An exploratory analysis was performed on all the investigated characteristics of the facilities (see Supplementary Table 1). Variables significantly associated with the outcome in the preliminary analysis were selected for subsequent modeling. An automated variable selection procedure was applied using a stepwise backward regression model with a removal threshold of 0.01 and a re-entry threshold of 0.05. The model included the following variables: geographical area, number of beds, receipt of information on procedures, difficulties in isolating and difficulties in transferring residents, staff shortages, denied or limited access to visitors, management of infected residents by facility medical staff, staff members testing positive, isolation of infected residents in rooms shared with other infected individuals, availability of register for physical restraints, and increased use of physical restraints, occurrence of adverse events, and increase in adverse events numbers.

Multicollinearity among variables was assessed before the stepwise procedure; a variance inflation factor (VIF) greater than 5 was considered indicative of problematic collinearity. The adequacy of the sample size for multivariable logistic regression was evaluated using the events-per-variable (EPV) criterion, with a minimum recommended threshold of 10 events per variable.

Data analysis was performed with STATA SE version 17 (Stata Corp LLC, College Station, TX, USA).

### 2.4 Ethical consent

On 27 February 2020, the Italian Presidency of the Council of Ministers authorized the collection and scientific dissemination of data concerning the COVID-19 pandemic by the ISS and other public health institutions (38). All procedures were performed in compliance with relevant laws and institutional guidelines. The study protocol was approved by the Ethics Committee of the Italian National Institute of Health (Protocol 0008790; 23 February 2023). No information on individual residents and staff members was collected. The privacy rights of human subjects were fully respected.

## 3 Results

Out of the 3,871 facilities that were contacted, 510 (13.2%) completed the initial part of the survey regarding their characteristics. Of these, 361, corresponding to 9.3% of the 3,871, completed the specific section of the questionnaire on COVID-19. These data correspond to 27,951 (23.3%) and 20,593 (17.1%) beds, respectively, out of the total 120,189 beds in the contacted facilities.

The 510 facilities that participated in the survey were substantially larger than the 3,361 that did not participate (54 vs. 28 beds, *p* < 0.001) and were less frequently located in the Southern macro-area (19% vs. 28%, *p* < 0.001).

### 3.1 Section 1: facility characteristics

Geographically, 345/510 (68%) facilities participating in the survey were located in the North, corresponding to 21,167 (75.7%) beds; 69 (13%) in the Center with 3,118 (11.2%) beds; and 96 (19%) in the South and Islands with 3,666 (13.1%) beds. Among the facilities, 99 (19.4%) were public, 285 (55.9%) were accredited private facilities, 122 (23.9%) were private, and 4 (0.8%) belonged to other categories. Residents were self-sufficient older adults in 221 (43.3%) facilities, non-self-sufficient older adults in 343 (67.3%), self-sufficient people with disabilities in 88 (17.2%), and non-self-sufficient people with disabilities in 171 (33.5%).

When comparing the facilities that filled out (*n* =361) and did not complete (*n* = 149) the second part of the questionnaire on the COVID-19 pandemic, the two groups were similar in terms of geographical distribution and the proportions of public vs. private RHOAD. However, they differed in the percentage of care-dependent residents, which was higher among non-responders (64.0% vs. 75.2%, *p* = 0.014).

### 3.2 Section 2: COVID-19 pandemic

**Figure 1:**
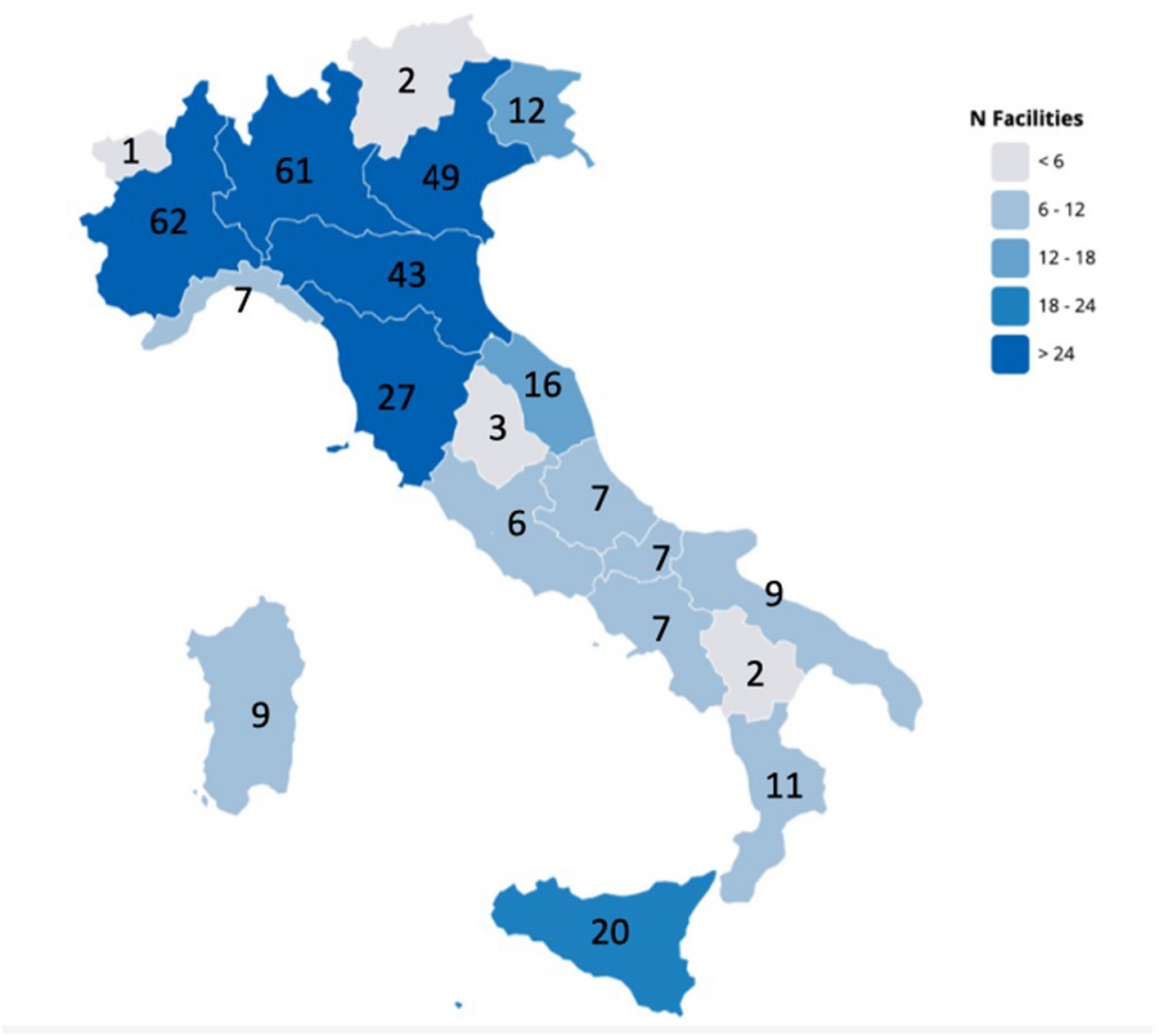
Distribution by region of 361 Italian RHOADs that responded to the COVID-19 pandemic survey.

Of the 361 facilities that filled out Section 2 of the survey (Figure 1), a group of 85 (23.5%), corresponding to 2,975 beds, reported no cases of infection in any of the four semesters of 2020–2021. A total of 189 (52.3%), corresponding to 11,102 beds, reported at least one case during the period analyzed. In addition, a group of 87 (24.2%) facilities, corresponding to 6,516 beds, did not provide data on the number of infected residents. A very small group, 11 out of 189 facilities with at least one case (5.8%), reported COVID-19 infections in each of the four semesters of 2020–2021; 10 of them were located in Northern Italy. Personnel involved in facility activities were mainly social and health workers (93.6%), with physicians representing the lowest proportion (9.1%).

Data on the temporal trend of the number of residents and COVID-19 infections are reported in Table 2. The percentages of residents who (i) tested positive for COVID-19, (ii) were hospitalized due to infection, and (iii) died in the facilities due to COVID-19 followed the same trend across the four semesters of 2020 and 2021. Namely, they increased from the first to the second semester of 2020 and then decreased until the end of 2021 (Table 2).

**Table 2:** Trend over 2020–2021 semesters in COVID-19 infection, hospitalization, and death rates among residents.

Data collected in the survey	1°semester 2020	2°semester 2020	1°semester 2021	2°semester 2021
a) N = 274 facilities providing the information				
Number of residents in the facilities				
Mean (min-max)	62 (1–555)	58 (2–550)	58 (2–565)	62 (2–602)
Median (IQR)	42 (16–76)	39 (15–72)	39 (15–72)	40 (16–79)
% of residents positive for COVID-19				
Mean (min-max)	11.0% (0%−100%)	16.8% (0%−100%)	12.8% (0%−100%)	7.4% (0%−100%)
% of residents hospitalized due to COVID-19				
Mean (min-max)	2.4% (0%−93%)	3.3% (0%−100%)	1.7% (0%−82%)	0.2% (0%−10%)
% of residents deceased in the facilities due to COVID-19				
Mean (min-max)	1.3% (0%−40%)	2.9% (0%−100%)	0.5% (0%−23%)	0.1% (0%−10%)
**b) N** =**189 subgroup of facilities with at least one case of COVID-19 in 2020–2021**				
Number of residents in the facilities				
Mean (min-max)	63 (1–455)	58 (6–430)	59 (6–443)	64 (4–452)
Median (IQR)	43 (18–80)	40 (16–74)	39 (17–75)	42 (18–80)
% of residents positive for COVID-19				
Mean (min-max)	15.6% (0%−100%)	24.1% (0%−100%)	18.3% (0%−100%)	10.6% (0%−100%)
% of residents hospitalized due to COVID-19				
Mean (min-max)	3.5% (0%−93%)	4.9% (0%−100%)	2.5% (0%−82%)	0.4% (0%−10%)
% of residents deceased in the facilities due to COVID-19				
Mean (min-max)	2.0% (0%−40%)	4.2% (0%−100%)	0.7% (0%−23%)	0.1% (0%−10%)

a) N = 274 facilities providing the information (Missing value 87, 24.2%), b) data from the subgroup of 189 facilities with at least one COVID-19 case in 2020–2021.

Compared to the facilities reporting no cases of infection (Table 3), those with at least one case of COVID-19 were more likely to be located in the North of Italy (*p* < 0.001), have had more beds (*p* < 0.001), and more often host non-self-sufficient people with disabilities (*p* = 0.049). Facilities with at least one COVID-19-positive resident more frequently had a physiotherapist on staff (*p* < 0.001). Moreover, these facilities more frequently reported receiving poor information on infection-containment procedures (*p* = 0.005) (Supplementary Table 1), experiencing staff shortages (*p* = 0.041), and facing difficulties in transferring COVID-19-infected residents to hospitals (*p* < 0.001), and isolating residents with suspected or confirmed COVID-19 infection (*p* = 0.001) in 2020. These differences remained significant in 2021, except for information on infection containment (*p* = 0.457) and staff shortages (*p* = 0.058).

**Table 3:** Comparison of characteristics between facilities with and without COVID-19 cases in 2020–2021.

Main facility characteristics	No cases *N* = 85	At least one case *N* = 189	*p*
Geographical macro-area			
North	37 (43.5%)	141 (74.6%)	**<0.001**
Centre	17 (20.0%)	26 (13.8%)	
South	31 (36.5%)	22 (11.6%)	
Type of facility			
Public	11 (12.9%)	36 (19.1%)	0.623
Accredited private	48 (56.5%)	104 (55.0%)	
Private	25 (29.4%)	47 (24.9%)	
Other	1 (1.2%)	2 (1.1%)	
Type of residents			
Self-sufficient older adult	37 (43.5%)	81 (42.9%)	0.892
Non-self-sufficient older adult	48 (56.5%)	114 (60.3%)	0.549
Self-sufficient people with disabilities	17 (20.0%)	29 (15.3%)	0.340
Non-self-sufficient people with disabilities	22 (25.9%)	72 (38.1%)	**0.049**
Total number of beds			
Media (min-max)	35 (2–121)	59 (6–368)	**<0.001**
Median (IQR)	24 (10–54)	45 (20–83)	
Workforce			
Physician	8 (9.4%)	17 (9.0%)	0.912
Psychologist	10 (11.8%)	39 (20.6%)	0.076
Nurse	70 (82.3%)	168 (88.9%)	0.138
Physiotherapist	48 (56.5%)	146 (77.3%)	**<0.001**
Social and health worker	79 (92.9%)	180 (95.2%)	0.439
Social welfare auxiliary	44 (51.8%)	112 (59.3%)	0.246
Social assistant	32 (37.6%)	67 (35.5%)	0.726
Educator/entertainer	75 (88.2%)	175 (92.6%)	0.238

Facilities for which information on COVID-19 cases was missing were excluded (87, 24.2%). Statistically significant multivariable results are indicated in bold.

Additionally, differences were observed regarding family visits: in facilities with no cases, family members and caregivers were more frequently denied entry (*p* < 0.001), whereas in facilities with at least one case, access was allowed but restricted to situations authorized by health management (*p* = 0.010). Furthermore, facilities that reported at least one case of COVID-19 more frequently reported: (i) SARS-CoV-2 test positivity among facility staff members (*p* < 0.001); (ii) management of the residents with COVID-19 infection, both suspected and confirmed, by the medical staff of the facilities (*p* = 0.023); (iii) isolation of suspected or confirmed cases in rooms with COVID-19 cohosting (*p* = 0.004); (iv) presence of a register for physical restraint (*p* = 0.022).

Concerning the use of restraints, facilities that reported at least one case of COVID-19 infection more frequently indicated an increase compared to 2019 than those without cases, both in 2020 (*p* = 0.029) and 2021 (*p* = 0.036). A difference was also found between the two groups in the occurrence of adverse events (*p* = 0.004) and in their increase during the study period (*p* = 0.014).

No significant differences were observed in flu and COVID-19 vaccination coverage between the two groups of facilities, with both achieving a coverage rate of 90%. More details are reported in Supplementary Table 1.

The multivariable analysis (Table 4) showed that being in the Center (OR 0.39, 95%CI 0.16–0.93) or in the South or Islands (OR 0.17, 0.07–0.39) was associated with a lower probability of having at least one COVID-19 case compared with those in the North.

**Table 4:** Factors associated with COVID-19 cases in 2020–2021: univariable and multivariable logistic regression analysis.

Variable related to the COVID-19 pandemic	Univariable				Multivariable			
	OR	95%CI		*p*	OR	95%CI		*p*
Geographical macro-area									
North	1.00				1.00			
Centre	0.40	0.20	0.82	0.012	**0.39**	**0.16**	**0.93**	**0.034**
South	0.19	0.10	0.36	<0.001	**0.17**	**0.07**	**0.39**	**<0.001**
Total number of beds									
0–20 (min-q1)	1.00				1.00			
21–44 (q1-median)	1.70	0.86	3.35	0.128	**2.29**	**1.05**	**4.97**	**0.037**
45–80 (median-q3)	1.73	0.88	3.37	0.110				
80+ (q3-max)	5.51	2.24	13.54	<0.001	**2.97**	**1.10**	**8.03**	**0.031**
Little information received about the procedures (yes vs. no)	2.24	1.28	3.94	0.005				
Staff shortage (yes vs. no)	1.71	1.02	2.87	0.042				
Difficulty in transferring (yes vs. no)	13.67	4.15	44.97	<0.001	**11.79**	**3.32**	**41.91**	**<0.001**
Difficulty in isolating (yes vs. no)	5.02	2.64	9.52	<0.001	**4.43**	**2.13**	**9.22**	**<0.001**
Access always denied to visitors (yes vs. no)	0.28	0.14	0.57	<0.001				
Limited access only to cases indicated (yes vs. no)	2.14	1.19	3.85	0.011	**2.16**	**1.06**	**4.38**	**0.033**
Facility's staff tested positive (yes vs. no)	6.87	3.00	15.70	<0.001				
Management of infected residents by facility medical staff (yes vs. no)	1.84	1.09	3.11	0.023				
Infected residents isolated in rooms shared with other infected residents (yes vs. no)	2.19	1.28	3.73	0.004				
Availability of a register for physical restraints (yes vs. no)	2.04	1.17	3.58	0.012				
Increase of physical restraints (yes vs. no)	2.92	1.16	7.31	0.022				
Occurrence of adverse events (yes vs. no)	2.79	1.49	5.24	0.001				
Increase in adverse events (yes vs. no)	4.23	1.44	12.45	0.009	**2.49**	**1.17**	**5.27**	**0.018**

OR, odds ratio; CI, 95% confidence interval; q1, first quartile; q3, third quartile. Statistically significant multivariable results are indicated in bold.

Furthermore, having a mean number of 80 or more beds increased almost threefold the risk for the facilities of having at least one COVID-19 case compared to facilities with up to 20 beds (OR 2.97, 1.10–8.03). Similarly, experiencing difficulties in transferring (OR 11.79, 3.32–41.91) and isolating (OR 4.43, 2.13–9.22) residents with suspected or confirmed COVID-19 infection in 2020 was positively associated with having had at least one case. Finally, facilities where more frequently the number of adverse events increased (OR 2.49, 1.17–5.27) and the access to visits was restricted (OR 2.16, 1.06–4.38) were confirmed to be associated in the multivariable analysis with the presence of at least one infection case.

## 4 Discussion

This national survey aimed to investigate the spread and impact of SARS-CoV-2 infection within Italian RHOAD and to identify the challenges these facilities faced during the pandemic. Since these facilities host vulnerable individuals (7, 8) and typically present conditions conducive to the spread of pathogens, such as numerous shared spaces, staff working in multiple facilities, and contact with outside individuals, the COVID-19 pandemic found fertile ground for its spread in these settings (3, 4). Alongside these factors, emergency preparedness in long-term care was insufficient, as several countries, including Italy, lacked public guidelines on infection control in LTC before 2020. Moreover, since Italy was one of the first countries affected, there was insufficient time to develop operational guidelines; the limited integration between the healthcare system and social services might have further contributed to the crisis experienced by these facilities (39). According to our survey, most RHOAD host older individuals who are not self-sufficient. However, a substantial portion of these facilities also accommodates non-self-sufficient individuals with disabilities, suggesting that these populations may be more vulnerable than the general population.

The RHOAD that responded to the survey were primarily located in Northern Italy, indicating an uneven distribution of both the facilities themselves and their accessibility in terms of bed availability. This finding aligns with other studies suggesting that social and healthcare services in Italy are predominantly situated in the North compared with the Central or Southern regions and the islands (29, 30, 40). This disparity persists despite differences in the prevalence of certain conditions. For example, although dementia prevalence is estimated to be higher in Northern Italy, the ratio of cases to available services (like daycare centers and nursing homes) continues to disadvantage the Central and Southern regions/Islands (33).

Analyzing the percentages of residents who tested positive for COVID-19, were hospitalized for COVID-19, or died in the facility due to COVID-19, there was an increase from the first to the second semester of 2020, followed by a decrease in the first semester of 2021, and a further reduction in the second semester of 2021. These changes are even more pronounced in the subgroup of facilities that recorded at least one case of COVID-19 during 2020–2021. This trend aligns with the Italian data from the Organization for Economic Co-operation and Development (41) and the European Observatory on Health Systems and Policies, which indicate that Italy was unable to reduce the infection curve in time. This resulted in the saturation of health services and a subsequent rise in infections and deaths. During the first wave (March–May 2020), Italy experienced the highest death rates in Europe (41). Mortality peaks were not homogeneous throughout the national territory, and were influenced by several factors such as mobility, demographic and geographical characteristics, population density, and hospital concentration. Northern Italy was initially the most impacted area, with excess mortality more than 50% higher than in the pre-pandemic period (42).

The decrease in death rates and excess mortality from 2020 to 2021 can be mostly explained by COVID-19 vaccination, which in Italy took place from December 2020 and initially targeted older adults (>80 years old), LTC residents, healthcare workers, and vulnerable people (43). At that time, Italy was no longer implementing a national lockdown, which ended on the 3rd of May 2020, but regional or provincial lockdowns were implemented according to the spread of the virus in the various areas. This most likely had a positive impact on the number of infected individuals, as indeed it did in the second half of 2020, but high vaccination coverage may have helped to maintain population health during the pandemic period (44). Additionally, it should be noted that the recorded data on hospitalizations and deaths due to COVID-19 are lower than those reported by a previous Italian survey on nursing homes (30). Although that study focused only on the first semester of the pandemic, and it is therefore not possible to make a long-term comparison, two different scenarios for these two types of structures had already emerged. The discrepancy between the Italian nursing homes, which experienced greater stress in this regard, and RHOAD may be related to differences in facility characteristics and resident profiles. Nursing homes tend to be healthcare facilities that provide support and care for people requiring assistance with activities of daily living and people with complex health problems (45).

We subsequently aimed to outline the profile of the RHOAD that reported at least one COVID-19 case among residents during the 2020–2021 period, in order to identify characteristics that may serve as protective or risk factors for the spread of epidemics. Specifically, beyond being located in the North, where viral circulation was higher as mentioned above, we found that specific facility characteristics, such as a larger number of beds or challenges in transferring or isolating residents with COVID-19 in 2020, were linked to an increased risk of having at least one COVID-19 case. Interestingly, the same results were reported by previous studies conducted in Italian and U.S. nursing homes (30, 46). Those studies also found an increased risk in facilities that reported a lack of PPE. Differently, our work showed no differences between facilities with or without COVID-19 cases regarding PPE shortages. One possible interpretation of the associations between certain variables and increased risk relates to the challenges of managing larger facilities or those with limited available spaces, which can hinder efforts to prevent contact between residents and complicate staff workflows. Previous studies have indeed reported that crowding in social or healthcare facilities is associated with increased risk of becoming infected or dying from COVID-19 or other infectious diseases (47–49). Since it was already known that LTC facilities often house vulnerable people and include shared areas, it is reasonable to assume that facilities with more residents experienced greater difficulty in effectively isolating and reducing contact. Referring to Benbow's model (50), which identifies engineering characteristics of LTC facilities that facilitate infection prevention, it can be hypothesized that, for the facilities that participated in our survey, differences between the facilities with and without COVID-19 cases could be attributable, beyond spatial separation, to the most basic elements of the pyramid, namely resident room zoning and the availability of private rooms.

Regarding the public or private nature of the facility, our analysis did not show significant differences, in line with other international studies highlighting the unclear and still poorly understood role of this variable. These differences may be rather attributable to organizational and structural characteristics of the facilities (39). Organizational and central management characteristics may have played a crucial role. In Europe, a standardized infection surveillance system for healthcare-associated infections in LTC facilities was lacking, likely due to limited resources allocated to these settings. The European Centre for Disease Prevention and Control established such a standardized system only at the onset of the pandemic (51). Moreover, as previously highlighted, many countries, including Italy, did not have specific protocols for the prevention and control of communicable diseases with a focus on LTC facilities (39). In contrast, Eastern countries, particularly Japan, a nation with high population density and a significantly aging population, had already implemented specific operational protocols before the pandemic, likely due to previous outbreaks such as SARS, influenza, and MERS. In countries with greater preparedness and response capacity, mortality rates in LTC facilities were comparatively lower (52).

Furthermore, we found that the facilities reporting at least one COVID-19 case among residents had applied the DPCM 08/05/2021 disposition less strictly, allowing family members or caregivers to visit the residents more frequently. Again, staff positivity to the SARS-CoV-2 test was reported more often in these facilities. These factors might relate to the variables mentioned above (contact with outside individuals and staff working in multiple facilities) that make the LTC setting particularly susceptible to viral spread (3, 4), but the cross-sectional nature of the study does not allow us to draw causative relations.

However, the reduction of social interactions raises ethical concerns. While limiting contact plays a key role in reducing viral spread and thus protecting individuals, especially those at higher risk of health complications, it also negatively affects mental health, increasing the risk of neuropsychiatric sequelae (28, 53, 54). In this regard, the term “psychopandemic” was coined precisely to describe the neuropsychiatric and psychopathological consequences of COVID-19 on mental health (55).

According to our survey data, the use of restraints increased by about 10% from 2019 to 2021, the use of psychotropic drugs by approximately 8%, and adverse events by 11.9%. Although the specific causes of these changes were not investigated, we can hypothesize that the psychological stress experienced by residents during this period contributed to these outcomes.

This work allowed us to delve into what was experienced by Italian RHOAD during the pandemic and to identify characteristics that helped facilities better cope with the difficulties arising from the spread of COVID-19. Collecting and analyzing these characteristics is useful for organizational and preventive purposes, aligning with the objectives of the NRRP, which aims to enhance the living conditions and social inclusion of older and non-self-sufficient individuals, and proposes alternative health models fostering person-centered care and continuity of care.

## 5 Limitations

There are some limitations to this work and to the interpretation of our results. The first limitation concerns the RHOAD sample that completed the questionnaire. Since participation in the survey was voluntary, we cannot exclude the possibility that the participating facilities were those most motivated or with the greatest organizational availability. Organizational problems could have been due to staff shortage or a still unstable epidemiological situation. This reduces the representativeness of our results.

Moreover, the study period considered predates the survey's administration, which could introduce potential recall bias. However, the survey questions required the consultation of administrative data and internal records, which should have minimized this bias and ensured the reliability of the responses. It should also be noted that the questionnaire did not collect detailed information on the diagnostic methods used to identify COVID-19 cases, which were defined at the national level and updated over time.

In addition, we collected data on the number of beds as an index of facility size. However, other structural indicators are relevant in infection prevention and containment, such as room sharing and the configuration of common areas (50). Indeed, some facilities may prefer single rooms or have a layout that can facilitate social distancing when needed. Future work on the spread of infections in RHOAD should consider these characteristics, in addition to the number of beds, in order to optimize a structural model of RHOAD that promotes resident health.

Future research should also delve into investigating the underlying causes of the increase in restraints, psychotropic drugs, and adverse events to explore the psychological impact experienced by people residing in RHOAD. It would also be important to investigate whether specific organizational or structural features may have modulated this impact to expand the health-promotion model that can be implemented in these facilities.

## 6 Conclusion

The RHOAD's risk of having at least one resident infected during the pandemic was modulated by specific facility characteristics. Particularly, geographic location, bed availability, and the possibility of transferring or isolating residents who tested positive were associated with the likelihood of having COVID-19 cases among residents. These results highlight the challenges faced by Italian RHOAD during the pandemic and help identify an organizational and structural model that could be more functional in promoting residents' wellbeing and in preparing for and managing future pandemics, which remain major public health concerns. This work is also relevant in light of the goals, actions, and commitments outlined in the NRRP, aiming at a person-centered model of care and continuity of care, as well as fostering inclusion and improvement of quality of life for older adults and people with disabilities.

## Group members of the Italian RHOAD Study Group

Abrate Mauro (Cambiano, TO), Agati Salvatore (Castel Di Iudica, CT), Aiello Alessandro (Borgetto, PA), Albanese Maria (Rizziconi, RC), Aldeghi Luca (Renate, MB), Alessio Laura (Asigliano Vercellese, VC), Alvisi Giuliano (Monterenzio, BO), Ambrosio Ilaria (Pontevico, BS), Angelino Remo (Pinerolo, TO), Arcangeli Lino (Ascoli Piceno, AP), Ardu Alessandra (Gonnosnò, OR), Arrobbio Cristiana (Poirino, TO), Arru Sara (Padru, SS), Arturi Serena (Salbertrand, TO), Ascheri Lorenzo (Borgonovo Val Tidone, PC), Astegiano Roberto (Cherasco, CN), Avantaggiato Clara (Fano, PU), Baesso Antonio (Torri Di Quartesolo, VI), Balbo Bruno (Ventimiglia, IM), Balbo Viola (Garessio, CN), Banfi Mirco (Barlassina, MB), Barba Calogero (Agrigento, AG), Barbara Bergo (San Secondo Di Pinerolo, TO), Barbero Valeria Monticello D'Alba, CN), Barbieri Virna (Pontevico, BS), Barone Gianmarco (Santa Marinella, RM), Barzaghi Alberto (Padova, PD), Basile Roberta (Torino, TO), Beccaria Antonella (Savigliano, CN), Bellini Maria Saveria (Colico, LC), Benigni Luca (Montemarciano, AN), Benini Davide (Ravenna, RA), Benzi Maria Rita (Farini, PC), Bernardello Davide (Sestri Levante, GE), Bernardi Katia (Cormons, GO), Berta Barbara (Chialamberto, TO), Berton Mirella (Treviso, TV), Bertossi Cristina (Tricesimo, UD), Bertuletti Giuseppe (Almenno San Salvatore, BG), Besutti Raffaele (Milano, MI), Bianco Roberta Rodello, CN), Binni Giacomo (Quarto D'Altino, VE), Biografi Arianna (Verucchio, RN), Bisconti Giovanni (Misilmeri, PA), Blanch Lorena (Gradisca D'Isonzo, GO), Bocca Daniela (Valenza, AL), Bocchini Cristina (Gatteo, FC), Bogani Valentina (Fagnano Olona, VA), Boggio Barbara (San Giusto Canavese, TO), Boggio Carla (Rivarolo Canavese, TO), Boldori Gianpaolo (Milano, MI), Bolondi Fabrizio (San Polo D'Enza, RE), Bolzoni Laura (Ornavasso, VB), Bonato Simona (Castelnuovo Del Garda, VR), Bonetti Maria Erika (Moncalieri, TO), Boni Ilaria (Borgo San Lorenzo, FI), Bonvicini Flaviano (Oppeano, VR), Boreggio Mario (Valdagno, VI), Borgna Franco (Pamparato, CN), Borraccia Valentina (Torino, TO), Borroni Silvia (Lesa, NO), Borsati Angelina (Mezzane Di Sotto, VR), Bortolin Carmela (Spinea, VE), Boscaro Federica (Montagnana, Cologna Veneta, PD), Boschetti Vittorio (Lendinara, RO), Bosco Luca (Vigasio, VR), Boscolo Fiore Elena (Chioggia, VE), Bosi Andrea (Lesmo, MB), Bosio Stefano (San Germano Chisone, TO), Bottazzoli Federico (Bibbona, LI), Botter Cinzia (Albino, BG), Botticella Maria Antonia (Dozza, BO), Bottone Daniela (Rivoli, TO), Bozzato Elisabetta (Padova, PD), Brach Loieta Laura (Bibiana, TO), Braghin Luisa (Codigoro, FE), Bragion Mariarosa (Quarrata, PT), Braida Rossella (Ticineto, AL), Brancato Giuseppe (Leni, ME), Brasile Raffaele (Cuorgnè, TO), Brianti Giancarlo (Udine, UD), Briccolani Debora (Imola, BO), Brugnoli Giulia (Ro, FE), Bruno Bianca (San Damiano Macra, CN), Bruno Fulvio (Schivenoglia, MN), Bruno Lorenza (San Salvo, CH), Bruno Luciano (Beinette, CN), Bruno Sandro (Tarcento, UD), Buccarella Valentina (Sogliano Al Rubicone, FC), Bulf Bruno (Taibon Agordino, BL), Bulgari Francesca (Cerro Maggiore, Legnano, MI), Bulleri Mariella (Empoli, FI), Buongiorno Adriana (Casalmaggiore, CR), Buoso Luca (Ceggia, VE), Cacciatori Stefano (San Giovanni Lupatoto, VR), Cafaro Ludovico (Zoppola, PN), Caimmi Michele Joris (Lainate, MI), Caldera Simona (Cavaglià, BI), Calicchio Giuseppe Nicola (Matera, MT), Camilli Meletani Serena (Fabriano, AN), Campanale Vito (Bernalda, MT), Candela Giuseppe (Saviano, NA), Cannavacciuolo Andrea (Udine, UD), Cannito Francesco (Bonate Sotto, BG), Capo Giuseppe (Salaparuta, TP), Cappannini Vincenzo (Citta' Della Pieve, PG), Cappelli Caterina (Marliana, PT), Capriotti Ines (Loreto, AN), Caramanico Luisa (Chieti, CH), Carlino Matteo (Riva Ligure, IM), Carnuccio Anna Maria (San Mauro Pascoli, FC), Caruso Mariacarmela (Avola, SR), Casalone Rinaldi Marta (Firenze, FI), Castagna Ilaria (Barbarano Vicentino, VI), Castoldi Davide (Besana In Brianza, MB), Catalano Ravaglioli Marta (Gaglianico, BI), Cattanei Mauretta (Pavia, PV), Cavion Barbara (Valli Del Pasubio, VI), Celentano Chiara (Torino, TO), Cencetti Alba (Riolo Terme, RA), Ceresa Paola Giuseppina (Sparone, Locana, TO), Chianese Valentina (Grosseto, GR), Chiaradia Giuseppe (Corigliano-Rossano, CS), Chiolo Luigi (Mazzarino, CL), Chittaro Loris (Venezia, VE), Cinelli Cinzia (Arezzo, AR), Ciocchini Valentina (Rozzano, MI), Cioni Sara (Bologna, BO), Cocco Chiara (Montecchio Maggiore, VI), Colamaio Giuseppe (Campobasso, Vinchiaturo, CB), Colombino Roberta (Cantalupa, TO), Colombo Lisa (Monza, MB), Colombo Sonia (Pontedassio, IM), Colturi Gennj (Tirano, SO), Coppola Cristina (Santa Maria Imbaro, CH), Cornaglia Paola (Cerrina, AL), Corsano Valentina (Vitulano, BN), Corsaro Maria (Misterbianco, CT), Covatta Maria Vittoria (Castelpetroso, IS), Craviolo Chiara (Mosso, BI), Crispino Giorgio (Mangone, Bocchigliero, CS), Cucchio Simona (Albese Con Cassano, CO), Curti Luisa (Peveragno, CN), Dagani Erika (Lodi, LO), Dal Mas Stefano (Fossalta Di Portogruaro, VE), Danzi Donatella (Castello Di Annone, AT), De Caris Rita (Monghidoro, BO), De Dea Valeria (Pieve Di Cadore, BL), De Faveri Piero (Conegliano, TV), De Leo Ilaria (Pordenone, PN), De Maria Mira M.T. (Mazzarino, CL), De Nale Paola (Chieri, TO), De Pieri Veronica (Trevignano, TV), De Rosa Massimo (Milano, MI), Debenedetti Federica (Loano, SV), Degiovanni Silvano (Casorzo, AT), Degl'Innocenti Marcello (Scarperia, FI), D'Elia Carlo (Venezia, VE), Desogus Cesare (Milis, OR), Di Cristofaro Carmelina (Campobasso, CB), Di Dolco Francesco (Roma, RM), Di Fazio Vincenzo (Castel Frentano, CH), Di Pietro Luisa (Montenero Di Bisaccia, CB), Di Santo Daniela (San Giovanni Teatino, CH), Di Stefano Chiavaro Vita (Piario, BG), Dogliani Maura (Cuneo, CN), Donati Silvia (Argenta, FE), Dotta Federica (Luserna San Giovanni, TO), Dotti Manuela (Darfo Boario Terme, BS), Dubinin Pietro Antonio (Caserta, CE), Duretto Cristina (Valperga, TO), Egidio Rodolfo (Benevento, BN), Ena Andrea (Sassari, SS), Enrico Gianluca (Andorno Micca, BI), Fabbrica Debora (Premilcuore, FC), Facci Francesco (Este, PD), Fariello Francesco (San Miniato, PI), Fenile Massimo (Acqui Terme, AL), Fera Achille (Girifalco, CZ), Feroldi Zaira (Rivoli, TO), Ferracin Roberta (Mondovì, CN), Ferrante Alfredo (Sondrio, SO), Ferrara Angelina (Paternopoli, AV), Ferrara Vito (Cassano Delle Murge, BA), Ferrari Massimo (Sorbolo, PR), Ferrari Michele (Parma, PR), Ferrario Fabio (Beregazzo Con Figliaro, CO), Fino Stefania (Verzuolo, CN), Fiorentini Samantha (Lariano, RM), Flaccadori Emanuele (Vigevano, PV), Fontana Solange Dominique (Parma, PR), Formentin Roberta (Barbarano Vicentino, VI), Fornaro Franca (Cumiana, TO), Franchi Patrizia (Bardi, PR), Frigo Beatrice (Vicenza, VI), Gabaldo Fabrizio (Casale Di Scodosia, PD), Gaiardoni Carlo (Villa Bartolomea, VR), Galaschi Donatella (Sale, AL), Galletta Giulia (Scilla, RC), Gallon Dario (Casale Monferrato, AL), Galluzzo Vincenza Cinzia (Aragona, AG), Gamberini Maria Giacinta (Modena, MO), Garzola Elena (Caresana, VC), Genetti Cristian (Cavour, TO), Gentile Simona Carmen Alessandra (Cremona, CR), Ghetti Greta (Lugo, RA), Ghio Daniela (Calcinato, BS), Ghio Giacomo (Boves, CN), Ghisellini Simona (Verona, VR), Giachino Daniela (Magliano Alfieri, CN), Giacon Marco (Montebelluna, TV), Giannoni Federica (Castelnuovo Val Di Cecina, PI), Giaretta Anna (Lonigo, VI), Giletta Giuseppe (Cavour, TO), Gillardo Mirko (Pareto, AL), Ginocchi M.Luisa (Borghetto Di Vara, SP), Giurca Gabriela Cristina (Montalto Delle Marche, AP), Gorga Caterina (Casarano, LE), Gori Maria Cristina (Terracina, LT), Gottardello Piera Antonia (Oleggio, NO), Grandini Alessandro (Rivoli, TO), Grasso Elisabetta (Genova, GE), Gravante Rosanna (Isernia, IS), Graziani Manuela (Rimini, RN), Grazzini Cristina (Vado Ligure, SV), Greco Giovanni (Carmiano, LE), Greco Maria (Ragusa, RG), Greco Rito (Catania, CT), Gregnanin Vanja (Giaveno, TO), Grigolon Lorenzo (Novellara, RE), Groppelli Marta (Saltrio, VA), Guidotti Lucia (Piancastagnaio, SI), Ianniello Giovanna (Bonea, BN), Iannolo Sara (Firenze, FI), Iannone Rosa (Roccabascerana, AV), Iemmi Stefania (San Polo D'Enza, RE), Kutin Anna (Gorizia, GO), Laganà Armenia (Cassano Magnago, VA), Lalli Vincenzo (Castiglione Messer Marino, CH), Lampreu Agnese (Sedilo, OR), Lanciotti Fabrizio (Savignano Irpino, AV), Landi Lara (Senigallia, AN), Lavatori Paolo (Ripe, AN), Leone Nicoletta (Milano, MI), Leoni Oriano (San Benedetto Val Di Sambro, BO), Leta Serena (San Mauro Castelverde, PA), Liberati Mario (Fermo, FM), Linguanti Sonia (Modica, RG), Lombardi Paola (Cigognola, PV), Lombardi Vincenzo (Bitritto, BA), Loni Veronica (Pisa, PI), Lorello Anna Elvira (Foligno, PG), Lucarini Patrizia (Cantiano, PU), Luciano Paola (Monterosso Grana, CN), Lulleri Sabrina (Siena, SI), Lupetti Ivana (Castiglion Fiorentino, AR), Madonia Carmelo (Cammarata, AG), Madrigali Marta (Lucca, LU), Magani Fabrizio (Muggiò, MB), Magni Sara (Paderno D'Adda, LC), Maiello Diletta (Ravenna, RA), Malagò Pier Maria (Comacchio, FE), Malinverni Pierpaolo (Vignola, MO), Mammola Aldo (Villanova Mondovì, CN), Mancinelli Elia (Belvedere Ostrense, AN), Mancini Maria Luisa (Marsciano, PG), Manfredi Francesco (Campegine, RE), Manoni Fiorella (Arezzo, AR), Marastoni Matteo (Quattro Castella, RE), Marceca Ivana (Borgetto, PA), Marchetto Roberta (Treviso, TV), Maresca Antonino (Meta, NA), Maria Ferrari (Castel Goffredo, MN), Mariani Gaetana (Tavernerio, CO), Marini Cecilia (Firenze, FI), Martineli Giulia (Milano, MI), Martini Maria (Dovadola, FC), Masala Daniela (Torino, TO), Masini Arturo (Portomaggiore, FE), Mason Silvana (Noale, VE), Massaro Clara (Milano, MI), Mastrodomenico Stefano (Godiasco, PV), Matina Antonio (Raffadali, AG), Matta Cecilia (Serravalle Scrivia, AL), Maurandi Giancarlo (Iglesias, CI), Mauro Orietta (Pavia Di Udine, UD), Mazzanti Monia (San Benedetto Val Di Sambro, BO), Mazzuca Mariaclaudia (Cento, FE), Mazzucchelli Sara (Roè Volciano, BS), Meggiolaro Gianmario (Caldiero, VR), Melenzio Nella (Airola, BN), Memoli Loredana (Soliera, MO), Menini Fabio (Melzo, MI), Mercantile Maria Luisa (Vicoforte, CN), Merella Waltere (Domusnovas, CI), Merletti Sergio (Cannobio, VB), Merra Salvatore (Roma, RM), Mesiti Rosa (Siderno, RC), Messori Elena (Campegine, RE), Mezzadra Davide (Cavriago, RE), Mezzadra Valeria (Mazzano, BS), Miccichè Giuseppe (Campobello Di Licata, AG), Mihalache Ana Maria (Prato, PO), Milano Brunello (Negrar, VR), Millo Eva (Trieste, TS), Mina Katia (Varese, VA), Mingrone Rosa (Duino-Aurisina, TS), Mondino Marco (Montemagno, AT), Mora Romina (San Gimignano, Poggibonsi, Colle Di Val D'Elsa, SI), Morcelli Valentino (Castello Dell'Acqua, SO), Moretti Lucia (Firenze, FI), Morici Moreno (Cupramontana, AN), Morrone Marco (Montalto Uffugo, CS), Moscato Brunetta (Signa, FI), Motta Santo (Ramacca, CT), Munari Diego (Brendola, VI), Muraca Luciano (Gizzeria, CZ), Mussner Erna (Ortisei, BZ), Nappi Carmine (Saviano, NA), Nasuti Emilio (Abbateggio, AQ), Nicolò Costantino (Teramo, TE), Nicosia Maria (Azeglio, TO), Niedermayr Kurt (Termeno Sulla Strada Del Vino, BZ), Nisticò Maria (Lanuvio, RM), Nizzoli Silvia (Reggio Nell'Emilia, RE), Notari Cleide (Reggio Nell'Emilia, RE), Novelli Andrea (Vedelago, TV), Nuti Federica (Forlimpopoli, FC), Oliverio Martina (Ferrara, FE), Orlandi Antonella (Bibbiena, AR), Ortolan Dania (Ponte Di Piave, TV), Pagliai Costanza (Firenze, FI), Paialunga Ivana (Ascoli Piceno, AP), Palermo Mario (Belvedere Marittimo, CS), Palmerio Vincenzo (Treglio, CH), Palmieri Carla (Fabriano, AN), Panu Ester Vittoria (Gassino Torinese, TO), Panzacchi Giacomo (Bologna, BO), Paolo Battocchio (Mel, BL), Pappacena Francesco (Parma, PR), Patella Luigi (Torremaggiore, FG), Paternò Lidia (Bologna, BO), Pecoraro Roberto (Capaccio, SA), Perina Valter (Verona, VR), Perna Alfonso (Crecchio, CH), Peruzzo Simona (Morsano Al Tagliamento, PN), Piazza Pierluigi (Parma, PR), Piccinni Liliana (Occhieppo Superiore, BI), Pina Francesca (Venezia, VC), Pinotti Marica (Agazzano, PC), Piscopo Anna (Albiate, MB), Pistillo Mirella (Castel Goffredo, MN), Pituello Matteo (Firenze, FI), Pizzo Elisabetta (Loiano, BO), Poggio Cinzia (Montechiaro D'Asti, AT), Poloni Daniela (Morengo, BG), Pompili Giuseppina (Modena, MO), Poppi Maura (Reggio Nell'Emilia, RE), Pozzobon Elisa (Venezia, VE), Prati Alfonso (Argenta, FE), Preiata Samuele (Castel San Pietro Terme, BO), Prezioso Alessandro (Casacalenda, CB), Puddu Laura (Cocquio-Trevisago, VA), Punnakkezhath Victoria (Ispica, RG), Quadretti Fabio Romano (Rovigo, RO), Quondam Girolami Donatella (Spoleto, PG), Racalbuto Giovanni (Palma Di Montechiaro, AG), Ragaglia Giampaolo (San Marcello, AN), Ramacciotti Daniele (Viareggio, LU), Ramello Cinzia (Alba, CN), Rao Donatella (Altavilla Milicia, PA), Rendina Enrico (Baceno, VB), Riggi Dalila (Caltanissetta, CL), Rizzo Tiziano (Crocetta Del Montello, TV), Roasio Silvano (Sanfront, CN), Rocco Lorella (Costanzana, VC), Rognoni Suor Natalina (Alessandria, AL), Romanò Stefano (Olgiate Comasco, Binago, CO), Romoli Vanessa (Ancona, AN), Rossetti Maria Pia (Monterubbiano, FM), Rossi Giorgio (Boves, CN), Rubin Lucia (Selvazzano Dentro, PD), Rudella Lorenzo (Breganze, VI), Ruffo Valentina (Pontenure, PC), Rusu Meluta Ecaterina (Montecassiano, MC), Saba Giorgia Valeria (Paderno Dugnano, MI), Sacchi Erica (Lodi, LO), Said Siwar (San Giovanni Al Natisone, UD), Salvicchi Rodolfo (Foiano Della Chiana, AR), Sandri Moira (Spilimbergo, PN), Santesso Paolo (Agordo, BL), Santoianni Alessandro (Gorizia, GO), Saraino Claudio (Procida, NA), Sartor Rosetta (Cormons, GO), Sassano Francesco (Monacilioni, CB), Sasso Flora (Lomello, PV), Scavone Vincenzo (Matera, MT), Schiavon Davide (Venezia, VE), Sciacione Silvia (Priverno, LT), Scirocco Giovanna (Castel Di Sasso, CE), Sciumbata Valentina (Cesena, FC), Scomparin Fabrizio (Cavasso Nuovo, PN), Sconosciuto Antonio (Massa, MS), Scotti Giovanni (Sospiro, CR), Scranni Simone (Cesena, FC), Sedda Sara (Bosa, OR), Segato Mauro Antonio (Santo Stino Di Livenza, VE), Senatori Silvia (Firenze, FI), Servodio Valentina (Milano, MI), Settembrini Vincenzo (Roseto Capo Spulico, CS), Setti Alessio (Samatzai, SU), Sibilla Salvatore (Guanzate, CO), Sicilia Antonio (Panni, FG), Signorile Massimiliano (Ortona, CH), Simon Simon Jesy (Novoli, LE), Sironi Stefano (Lissone, MB), Soave Daniela (Arluno, MI), Sora Elena (Bagnolo San Vito, MN), Spadone Raffaella (Canzo, CO), Stanga Valentina (Brescia, BS), Stecco Francesca (Varese, VA), Stefanin Cristina (Vanzago, MI), Stortolani Stefano (Padova, PD), Succu Mariaflora (Firenze, FI), Taccardi Riccardo (Minervino Murge, BT), Tacchino Di Girolamo Emanuela (Genova, GE), Tardino Rosa Maria (Prato, PO), Tardivo Marta (Villadose, RO), Thomas Anneyamma (Cesena, FC), Titon Fabrizia (San Pietro Al Natisone, UD), Tiziani Tiziana (Buriasco, TO), Todaro Biagio (Capo D'Orlando, ME), Tola Rita Erina (Villanova Truschedu, OR), Toniolo Alberto (San Giorgio Delle Pertiche, PD), Tonon Monica (Meduna Di Livenza, Motta Di Livenza, TV), Toppano Paola (Tricesimo, UD), Torti Claudia (Pontecurone, AL), Tosi Silvia Tosi Silvia (Modena, MO), Trattenero Chiara (Valdagno, VI), Trezzolani Alessandra (Caprino Veronese, VR), Tronchin Marco (Mogliano Veneto, TV), Trucchi Giuseppe (Imperia, IM), Tuninetto Claudio (Villafranca Piemonte, TO), Uliana Maria Aurora (Zero Branco, TV), Vadori Marcello (Brescia, BS), Valdambrini Elisa (Pistoia, PT), Valentini Arnaldo Rodolfo (Lecce, San Pietro In Lama, Maglie, LE), Valori Virna (Corinaldo, AN), Vandini Desolina (Ferrara, FE), Vanzetti Adele (Challand-Saint-Anselme, AO), Vazzana Angelo (Vestignè, TO), Veggetti Marina (Pozzo D'Adda, MI), Venturini Nicole (Villasanta, MB), Verdolin Dino (Arcole, VR), Vigato Federico (Genova, GE), Vignoli Massimiliano (San Marcello Pistoiese, PT), Vignoli Valeria (Fontanelice, Borgo Tossignano, BO), Visigalli Andrea (Castelverde, CR), Vitali Nicoletta (Cotignola, RA), Vito Giuseppe (Bologna, BO), Vottero Sergio (Piscina, TO), Zago Paola (Biella, BI), Zambelli Angela (Crespino, RO), Zampicinini Franco (Castelnuovo Don Bosco, AT), Zanca Pamela e Merli Federica (Riccione, RN), Zanini Annalisa (Soncino, CR), Zannoni Daniela (Meldola, FC), Zanotti Luigi (Capriate San Gervasio, BG), Zanzanaini Paola (Carrara, MS), Ziella Jonathan (Vanzago, MI), Zigliani Roberta (Hone, AO), Zingale Valeria (Orvieto, TR), Zoccarato Cinzia (Castelnuovo Calcea, AT).

## Data Availability

The raw data supporting the conclusions of this article will be made available by the authors, without undue reservation.
