# Supplementary material for: A national survey on COVID-19 infection in Italian retirement homes for older adults and persons with disabilities
Source: Front Public Health. 2026 Jun 30;14:1872101. doi: 10.3389/fpubh.2026.1872101 (PMC13365346; doi:10.3389/fpubh.2026.1872101)
Supplement: Supplementary file 1 [file Table_1.DOCX]

**Supplementary Table 1.** COVID-19 data expressed as N (%) or median (interquartile range - IQR) and mean (min-max).

* Total N=361 (including 87 facilities with missing data on the number of COVID-19 infections).

| ***Second section of the questionnaire: COVID-19 Pandemic*** | **Total***  **N=361** | **No case**  **N=85** | **At least 1 case**  **N=189** | ***p*** |
| --- | --- | --- | --- | --- |
| Difficulties experienced in 2020, n(%) |  |  |  |  |
| Little information received about the procedures | 139 (38.5%) | 22 (25.9%) | 83 (43.9%) | **0.005** |
| Lack of medications | 26 (7.2%) | 2 (2.3%) | 15 (7.9%) | 0.076 |
| Lack of Personal Protective Equipment | 223 (61.8%) | 54 (63.5%) | 127 (67.2%) | 0.553 |
| Staff shortage | 187 (51.8%) | 35 (41.2%) | 103 (54.5%) | **0.041** |
| Difficulty in transferring residents affected by COVID-19 to hospitals | 76 (21.1%) | 3 (3.5%) | 54 (28.6%) | **<0.001** |
| Difficulties in isolating residents affected by Covid-19 | 121 (33.5%) | 13 (15.3%) | 79 (41.8%) | **<0.001** |
| Difficulties experienced in 2021, n(%) |  |  |  |  |
| Little information received about the procedures | 28 (7.8%) | 5 (5.9%) | 16 (8.5%) | 0.457 |
| Lack of medications | 10 (2.8%) | 1 (1.2%) | 5 (2.7%) | 0.442 |
| Lack of Personal Protective Equipment | 48 (13.3%) | 15 (17.7%) | 23 (12.2%) | 0.225 |
| Staff shortages | 192 (53.2%) | 39 (45.9%) | 110 (58.2%) | 0.058 |
| Difficulty in transferring residents affected by COVID-19 to hospitals | 48 (13.3%) | 3 (3.5%) | 35 (18.5%) | **0.001** |
| Difficulties in isolating residents affected by Covid-19 | 110 (30.5%) | 14 (16.5%) | 70 (37.0%) | **0.001** |
| How was the Prime Ministerial Decree of 05/08/2021 relating to visits by family members/caregivers applied? (more than one answer allowed), n(%) |  |  |  |  |
| Access was always denied to family members/caregivers | 51 (14.1%) | 21 (24.7%) | 16 (8.5%) | **<0.001** |
| Access has been limited only to cases indicated by the health management of the facility | 122 (33.8%) | 19 (22.3%) | 72 (38.1%) | **0.010** |
| Visits from relatives and volunteers were ensured to avoid the consequences on residents’ health due to prolonged isolation | 87 (24.1%) | 17 (20.0%) | 51 (27.0%) | 0.216 |
| Visits were allowed under safe conditions through adequate protective equipment and environmental conditions | 252 (69.8%) | 60 (70.6%) | 146 (77.2%) | 0.238 |
| In compliance with the safety measures and taking into account the epidemiological context of the geographical area, opportunities to go outside the residence were authorized | 120 (33.2%) | 29 (34.1%) | 69 (36.5%) | 0.703 |
| The normal visit routine was restored | 301 (83.4%) | 66 (77.6%) | 161 (85.2%) | 0.126 |
| Have alternative forms of communication with family members/caregivers been adopted to the facility? N (%) |  |  |  |  |
| Videocalls | 328 (90.9%) | 79 (92.9%) | 182 (96.3%) | 0.468 |
| Phone calls | 332 (92.0%) | 80 (94.1%) | 184 (97.3%) | 0.186 |
| Has the facility's staff ever tested positive for SARS-CoV-2?, n (%) | 314 (88.4%) | 63 (74.1%) | 177 (93.6%) | **<0.001** |
| Has a written management plan/procedure/protocol been developed for residents with COVID-19 (suspected or confirmed)?, n(%) | 340 (95.8%) | 82 (96.5%) | 185 (97.9%) | 0.325 |
| Has *ad hoc* counseling been received for clinical management and/or prevention and control of COVID-19?, n(%) | 227 (64.1%) | 55 (64.7%) | 130 (68.8%) | 0.650 |
| The management of the resident with COVID-19 (suspected or confirmed) wass carried out by, n(%) |  |  |  |  |
| General Practitioner | 203 (56.2%) | 45 (52.9%) | 118 (62.4%) | 0.139 |
| Medical staff of the facility | 182 (50.4%) | 31 (36.5%) | 97 (51.3%) | **0.023** |
| External consultants | 51 (14.1%) | 14 (16.5%) | 31 (16.4%) | 0.989 |
| Is it possible to isolate residents with a confirmed or suspected diagnosis of COVID-19, n(%) |  |  |  |  |
| No | 9 (2.5%) | 7 (3.4%) | 1 (1.5%) | 0.051 |
| Yes, single room | 251 (69.5%) | 59 (69.4%) | 136 (72.0%) | 0.667 |
| Yes, a room shared with other residents with COVID-19 | 239 (66.2%) | 47 (55.3%) | 138 (73.0%) | **0.004** |
| Yes, transfer to a dedicated facility | 13 (3.6%) | 4 (4.7%) | 7 (3.7%) | 0.696 |
| Yes, otherwise specify | 28 (7.8%) | 6 (7.1%) | 16 (8.5%) | 0.692 |
| Is the facility equipped with a register for physical restraints and their monitoring?, n(%) |  |  |  |  |
| No | 90 (24.9%) | 35 (41.2%) | 47 (24.9%) | **0.022** |
| Yes | 215 (59.6%) | 43 (50.6%) | 118 (62.4%) |  |
| Not available | 56 (15.5%) | 7 (8.2%) | 24 (12.7%) |  |
| Number physical restraints 2020-2021, median (IQR) | 17 (5-55) | 17 (4-31) | 19 (6-55) | 0.334 |
| Has an increase in restraints been detected in 2020 compared to 2019?, n(%) |  |  |  |  |
| No | 248 (68.7%) | 74 (87.1%) | 139 (73.5%) | **0.029** |
| Yes | 27 (7.5%) | 2 (2.3%) | 18 (9.5%) |  |
| Not available | 86 (23.8%) | 9 (10.6%) | 32 (16.9%) |  |
| Has an increase in restraints been detected in 2021 compared to 2019?, n(%) |  |  |  |  |
| No | 236 (65.4%) | 71 (83.5%) | 131 (69.3%) | **0.036** |
| Yes | 39 (10.8%) | 5 (5.9%) | 28 (14.8%) |  |
| Not available | 86 (23.8%) | 9 (10.6%) | 30 (15.9%) |  |
| When did you record the greatest number of restraints?, n(%) |  |  |  |  |
| 2020 | 62 (17.2%) | 13 (15.3%) | 40 (21.2%) | 0.172 |
| 2021 | 59 (16.3%) | 12 (14.1%) | 38 (20.1%) |  |
| Not available | 240 (66.5%) | 60 (70.6%) | 111 (58.7%) |  |
| Compared to 2019, did you detect an increase in the use of psychotropic drugs in 2020/2021? n(%) |  |  |  |  |
| No | 230 (63.7%) | 69 (81.2%) | 132 (69.8%) | 0.124 |
| Yes | 31 (8.6%) | 7 (8.2%) | 20 (10.6%) |  |
| Not available | 100 (27.7%) | 9 (10.6%) | 37 (19.6%) |  |
| If yes, which  Benzodiazepines | 16 (4.4%) | 3 (3.5%) | 10 (5.3%) | 0.526 |
| Antidepressants | 19 (5.3%) | 3 (3.5%) | 13 (6.9%) | 0.274 |
| Anti psychotics | 20 (5.5%) | 6 (7.1%) | 13 (6.9%) | 0.957 |
| Did you record any adverse events?, n(%) |  |  |  |  |
| No | 182 (50.4%) | 61 (71.8%) | 97 (51.3%) | **0.004** |
| Yes | 103 (28.5%) | 16 (18.8%) | 71 (37.6%) |  |
| Not available | 76 (21.1%) | 8 (9.4%) | 21 (11.1%) |  |
| If yes, specify the number (median (IQR)): |  |  |  |  |
| -Adverse events involving staff only | 2.6 (0-100) | 1.1 (0-6) | 3.1 (0-100) | 0.732 |
| - Adverse events involving residents only | 30.2 (0-681) | 25.9 (0-167) | 30.7 (1-681) | 0.550 |
| - Adverse events involving both staff and residents | 5.7 (0-120) | 5.1 (0-40) | 6.0 (0-120) | 0.265 |
| Did you register an increase in adverse events in 2020/2021 compared to previous years?, n(%) |  |  |  |  |
| No | 239 (66.2%) | 72 (84.7%) | 132 (69.8%) | **0.014** |
| Yes | 43 (11.9%) | 4 (4.7%) | 31 (16.4%) |  |
| Not available | 79 (21.9%) | 9 (10.6%) | 26 (13.8%) |  |
| Has a staff training and assistance program with practical exercises been carried out specifically for COVID 19?, n(%) |  |  |  |  |
| No | 24 (6.7%) | 5 (5.9%) | 14 (7.4%) | 0.897 |
| Yes | 318 (88.1%) | 79 (92.9%) | 173 (91.5%) |  |
| Not available | 19 (5.3%) | 1 (1.2%) | 2 (1.1%) |  |
| Has a staff training and assistance program on the correct use of personal protective equipment been carried out?, n(%) |  |  |  |  |
| No | 6 (1.7%) | 1 (1.2%) | 4 (2.1%) | 0.688 |
| Yes | 340 (94.2%) | 84 (98.8%) | 184 (97.4%) |  |
| Not available | 15 (4.2%) | 0 (0%) | 1 (0.5%) |  |
| Have initiatives been taken to raise residents' awareness regarding the prevention and control of Covid-19?, n(%) |  |  |  |  |
| No | 9 (2.5%) | 2 (2.3%) | 7 (3.7%) | 0.814 |
| Yes | 330 (91.4%) | 82 (96.5%) | 179 (94.7%) |  |
| Not available | 22 (6.1%) | 1 (1.2%) | 3 (1.6%) |  |
| Are hydroalcoholic gel dispensers available to staff in the facility? n(%) |  |  |  |  |
| No | 1 (0.3%) | 0 (0%) | 1 (0.5%) | 0.636 |
| Yes | 350 (97.0%) | 85 (100%) | 187 (98.9%) |  |
| Not available | 10 (2.8%) | 0 (0%) | 1 (0.5%) |  |
| Has the obligation to use masks been adopted to combat COVID-19 infection?, n(%) | 345 (95.6%) | 84 (98.8%) | 185 (97.9%) | 0.591 |
| Indicate the percentage of flu vaccination coverage of residents in December 2022, mean (min-max) | 89%  (0%-100%) | 90%  (6%-100%) | 89%  (0%-100%) | 0.070 |
| Indicate the percentage of COVID-19 vaccination coverage of residents in December 2022, mean (min-max) | 95%  (3%-100%) | 92%  (3%-100%) | 96%  (9%-100%) | 0.323 |
