# Supplementary material for: A national survey on COVID-19 infection in Italian retirement homes for older adults and persons with disabilities
Source: Front Public Health. 2026 Jun 30;14:1872101. doi: 10.3389/fpubh.2026.1872101 (PMC13365346; doi:10.3389/fpubh.2026.1872101)
Supplement: Supplementary file 2 [file Table_2.DOCX]

**Questionnaire**

***Section 1:* *facilities characteristics***

1. Date of compilation:

2. Facility name:

3. Type of facility : Public [ ]; Accredited private [ ]; Private [ ]; Other[ ]

Specify: _______________________

4. Characteristics of residents:

| Self-sufficient older people [ ];  Non-self-sufficient older people [ ];  Self-sufficient people with disabilities [ ];  Non-self-sufficient people with disabilities [ ];  Other (specify:___) |
| --- |
|  |

5. Region:

6. Province:

7. Municipality:

8. Address:

9. Telephone number(s):

10. Email address:

11. Website:

12. Opening days to visitors:

13. Opening hours to visitors:

14. Local health authority (affiliation):

**Facility manager**

Name and surname:

Mobile:

Email address:

I have read the Privacy Policy and authorize the processing of my personal data according to Regulation (EU) 2016/679

***Section 2: COVID-19 Pandemic***

1.What kind and how many professionals work in the facility?

- Nurse, indicate number: [ ]

- Social health worker, indicate number: [ ]

- Social welfare auxiliary, indicate number: [ ]

- Social worker, indicate number: [ ]

- Physiotherapist, indicate number: [ ]

- Educator/entertainer, indicate number: [ ]

- Cultural mediator, indicate number: [ ]

- Other staff who may have close contact with residents (e.g. podiatrist, hairdresser, kitchen staff, etc.), specify:

___________________indicate number: [ ]

___________________indicate number: [ ]

___________________indicate number: [ ]

2. How many beds are there in the facility?

Number: [ ]

3. How many residents were present in the facility?

from 1 January 2020 to 30 June 2020; Number: [ ]

from 1 July 2020 to 31 December 2020; Number: [ ]

from 1 January 2021 to 30 June 2021; Number: [ ]

from 1 July 2021 to 31 December 2021; Number: [ ]

4. How many residents tested positive for Covid-19?

from 1 January 2020 to 30 June 2020; Number: [ ]

from 1 July 2020 to 31 December 2020; Number: [ ]

from 1 January 2021 to 30 June 2021; Number: [ ]

from 1 July 2021 to 31 December 2021; Number: [ ]

5. How many residents were hospitalized?

from 1 January 2020 to 30 June 2020; Number: [ ]

from 1 July 2020 to 31 December 2020; Number: [ ]

from 1 January 2021 to 30 June 2021; Number: [ ]

from 1 January 2020 to 30 December 2021; Number: [ ]

6. How many residents were hospitalized for Covid-19?

from 1 January 2020 to 30 June 2020; Number: [ ]

from 1 July 2020 to 31 December 2020; Number: [ ]

from 1 January 2021 to 30 June 2021; Number: [ ]

from 1 January 2020 to 30 December 2021; Number: [ ]

7. How many residents died in the facility?

from 1 January 2020 to 30 June 2020; Number: [ ]

from 1 July 2020 to 31 December 2020; Number: [ ]

from 1 January 2021 to 30 June 2021; Number: [ ]

from 1 July 2021 to 31 December 2021; Number: [ ]

8. How many residents died for Covid-19 in the facility?

from 1 January 2020 to 30 June 2020; Number: [ ]

from 1 July 2020 to 31 December 2020; Number: [ ]

from 1 January 2021 to 30 June 2021; Number: [ ]

from 1 July 2021 to 31 December 2021; Number: [ ]

9. What were the main difficulties experienced by the facility during the COVID-19 Pandemic in 2020?

(more than one answer allowed)

a. Little information received about the procedures to be carried out to control the spreading of the infection [ ]

b. Lack of medications [ ]

c. Lack of Personal Protective Equipment [ ]

d. Staff shortage [ ]

e. Difficulty in transferring residents affected by COVID-19 to hospitals [ ]

f. Difficulties in isolating residents affected by Covid-19 [ ]

g. Other (specify): _________________________________________________

10. What were the main difficulties experienced by the facility during the COVID-19 Pandemic in 2021?

(more than one answer allowed)

a. Little information received about the procedures to be carried out to control the spreading of the infection [ ]

b. Lack of medications [ ]

c. Lack of Personal Protective Equipment [ ]

d. Staff shortage [ ]

e. Difficulty in transferring residents affected by COVID-19 to hospital facilities [ ]

f. Difficulties in isolating residents affected by Covid-19 [ ]

g. Other (specify): _________________________________________________

11. How was the Prime Ministerial Decree of 05/08/2021 relating to visits by family members/caregivers applied? (more than one answer allowed)

- The access was always denied to family members/caregivers [ ]

- The access has been limited only to cases indicated by the health management of the facility [ ]

- Visits from relatives and volunteers have been ensured to avoid the consequences on residents’ health due to prolonged isolation [ ]

- The visits were possible due to safety conditions through adequate protective equipment and environmental conditions [ ]

- In compliance with the safety measures and taking into account the epidemiological context of the geographical area of reference, opportunities to go outside the residence have been authorized [ ]

11.1 The normal visit routine has been restored:

NO [ ]

YES [ ] if yes, since when? ______________

12. Have alternative forms of communication with family members/caregivers been adopted to the facility?

- NO [ ]

- YES [ ]

- NOT AVAILABLE [ ]

If yes, indicate the date since these measures were adopted: DD/MM/YYYY

What forms have been adopted:

- video calls NO [ ] YES [ ] NOT AVAILABLE [ ]

- phone calls NO [ ] YES [ ] NOT AVAILABLE [ ]

- Other (specify): _____________________________________________________

13. Has the facility's staff ever tested positive for SARS-CoV-2?

- NO [ ]

- YES [ ]

- NOT AVAILABLE [ ]

14. Has a written management plan/procedure/protocol been developed for the residents with COVID-19 (suspected or confirmed)?

- NO [ ]

- YES [ ]

- NOT AVAILABLE [ ]

15. Has *ad hoc* counseling been received for clinical management and/or prevention and control of COVID-19?

- NO [ ]

- YES [ ] (specify the type of counseling): _________________________________________

- NOT AVAILABLE [ ]

16. The management of the resident with COVID-19 (suspected or confirmed) is carried out by:

a. General Practitioner [ ]

b. Medical staff of the facility [ ]

c. External consultants [ ]

d. Other (specify): ______________________________________________

17. Is it possible to isolate residents with a confirmed or suspected diagnosis of COVID-19?

(possibility to indicate multiple options)

- NO [ ]

- YES (single room) [ ]

- YES (room with other residents with COVID-19) [ ]

- YES (transfer to a dedicated structure) [ ]

- YES (otherwise specify): __________________________________________________

18. Is the facility equipped with a register for physical restraint and its monitoring?

*Physical restraint has been defined as "any action or procedure that prevents the movement of a person's free body to a position of choice and/or normal access to his or her body by the use of any method attached to or adjacent to the body of a person whom he/she cannot easily control or remove”*

- NO [ ]

- YES [ ]

- NOT AVAILABLE [ ]

if yes, enter the number of restraints in the period 2020-2021[ ]

19. Was there an increase in restraints in 2020 compared to 2019?

- NO [ ]

- YES [ ]

- NOT AVAILABLE [ ]

20. Has an increase in restraints been detected in 2021 compared to 2019?

- NO [ ]

- YES [ ]

- NOT AVAILABLE [ ]

21. When did you record the greatest number of restraints?

-2020 [ ]

2021 [ ]

- NOT AVAILABLE [ ]

2 2. Compared to 2019, did you detect an increase in the use of psychotropic drugs in 2020/2021? (i.e., benzodiazepines, antidepressants, antipsychotics)

- NO [ ]

- YES [ ]

- NOT AVAILABLE [ ]

If yes, specify for which type of drug:

- benzodiazepines [ ]

- antidepressants [ ]

- antipsychotics [ ]

23. Did you record any adverse events?

*Adverse events might present as falls, injuries, emotional distress and behavioral disturbances, delirium, adverse drug events, dehydration, and intestinal obstruction*

- NO [ ]

- YES [ ]

- NOT AVAILABLE [ ]

If yes, specify **the number of:**

- Adverse events involving staff only: [ ]

- Adverse events involving residents only: [ ]

- Adverse events involving both staff and residents: [ ]

24. Did you register an increase in adverse events in 2020/2021 compared to previous years?

- NO [ ]

- YES [ ]

- NOT AVAILABLE [ ]

25. Has a staff training and assistance program with practical exercises been carried out specifically for COVID 19? (Italian National Institute of Health FAD course, video, webinar...)

- NO [ ]

- YES [ ]

- NOT AVAILABLE [ ]

26. Has a staff training and assistance program on the correct use of personal protective equipment been carried out?

- NO [ ]

- YES [ ]

- NOT AVAILABLE [ ]

27. Have initiatives been taken to raise residents' awareness regarding the prevention and control of Covid -19?

- NO [ ]

- YES [ ]

- NOT AVAILABLE [ ]

28. Are hydroalcoholic gel dispensers available to staff in the facility?

- NO [ ]

- YES [ ]

- NOT AVAILABLE [ ]

29. What measures have been adopted and/or exist to prevent the spread of COVID-19 infection?

- Mandatory use of face masks [ ]

- Other (specify)_________________________________________________

30. Indicate the percentage of vaccination coverage of residents in December 2022

- flu vaccine: [ ] NOT AVAILABLE [ ]

- anti-Covid vaccine: [ ] NOT AVAILABLE [ ]

Thanks for your collaboration
